# Management of hemodynamically unstable pelvic trauma: results of the first Italian consensus conference (cooperative guidelines of the Italian Society of Surgery, the Italian Association of Hospital Surgeons, the Multi-specialist Italian Society of Young Surgeons, the Italian Society of Emergency Surgery and Trauma, the Italian Society of Anesthesia, Analgesia, Resuscitation and Intensive Care, the Italian Society of Orthopaedics and Traumatology, the Italian Society of Emergency Medicine, the Italian Society of Medical Radiology -Section of Vascular and Interventional Radiology- and the World Society of Emergency Surgery)

**DOI:** 10.1186/1749-7922-9-18

**Published:** 2014-03-07

**Authors:** Stefano Magnone, Federico Coccolini, Roberto Manfredi, Dario Piazzalunga, Roberto Agazzi, Claudio Arici, Marco Barozzi, Giovanni Bellanova, Alberto Belluati, Giorgio Berlot, Walter Biffl, Stefania Camagni, Luca Campanati, Claudio Carlo Castelli, Fausto Catena, Osvaldo Chiara, Nicola Colaianni, Salvatore De Masi, Salomone Di Saverio, Giuseppe Dodi, Andrea Fabbri, Giovanni Faustinelli, Giorgio Gambale, Michela Giulii Capponi, Marco Lotti, GianMariano Marchesi, Alessandro Massè, Tiziana Mastropietro, Giuseppe Nardi, Raffaella Niola, Gabriela Elisa Nita, Michele Pisano, Elia Poiasina, Eugenio Poletti, Antonio Rampoldi, Sergio Ribaldi, Gennaro Rispoli, Luigi Rizzi, Valter Sonzogni, Gregorio Tugnoli, Luca Ansaloni

**Affiliations:** 1 First General Surgery Unit, Ospedale Papa Giovanni XXIII, Bergamo, Italy; 2 Interventional Radiology, Ospedale Papa Giovanni XXIII, Bergamo, Italy; 3 Emergency Medicine Unit, Ospedale Papa Giovanni XXIII, Bergamo, Italy; 4 Second General Surgery, Ospedale Santa Chiara, Trento, Italy; 5 Orthopedics and Traumatology, Presidio Ospedaliero di Ravenna, Ravenna, Italy; 6 Intensive Care Unit, Ospedali Riuniti di Trieste, Trieste, Italy; 7 Professor of Surgery University of Colorado, Denver Health Medical Center, Denver, USA; 8 Orthopedics and Traumatology, Ospedale Papa Giovanni XXIII, Bergamo, Italy; 9 Emergency Surgery, Azienda Ospedaliera Universitaria, Parma, Italy; 10 General Surgery-Trauma Team, Ospedale Niguarda Ca’ Granda, Milan, Italy; 11 Epidemiology, Ospedale Meyer, Florence, Italy; 12 Trauma Surgery and General and Emergency Surgery, Ospedale Maggiore, Bologna, Italy; 13 Clinica Chirurgia, University of Padova, Padova, Italy; 14 Emergency Medicine, Ospedale Morgagni-Pierantoni, Forlì, Italy; 15 Intensive Care and Anesthesiology, Ospedale Morgagni-Pierantoni, Forlì, Italy; 16 Intensive Care Unit, Ospedale Papa Giovanni XXIII, Bergamo, Italy; 17 Orthopedics and Traumatology, Azienda Ospedaliera-Universitaria S. Luigi Gonzaga Orbassano - University of Torino, Torino, Italy; 18 Shock and Trauma, Ospedale San Camillo – Forlanini Roma, Rome, Italy; 19 Interventional Radiology, Ospedale Cardarelli, Naples, Italy; 20 Interventional Radiology, Ospedale Niguarda Ca’ Granda, Milan, Italy; 21 Compartimental Sindrome Unit, Policlinico Umberto I, Rome, Italy; 22 General Surgery, Azienda Sanitaria Locale Na1 Centro Presidio Ospedaliero Ascalesi, Naples, Italy; 23 Anesthesiology Service, Ospedale Papa Giovanni XXIII, Bergamo, Italy; 24 Trauma Surgery, Ospedale Maggiore, Bologna, Italy

**Keywords:** Pelvic trauma, Angiography, Preperitoneal pelvic packing, External fixation, Pelvic binder

## Abstract

Hemodynamically Unstable Pelvic Trauma is a major problem in blunt traumatic injury. No cosensus has been reached in literature on the optimal treatment of this condition. We present the results of the First Italian Consensus Conference on Pelvic Trauma which took place in Bergamo on April 13 2013. An extensive review of the literature has been undertaken by the Organizing Committee (OC) and forwarded to the Scientific Committee (SC) and the Panel (JP). Members of them were appointed by surgery, critical care, radiology, emergency medicine and orthopedics Italian and International societies: the Italian Society of Surgery, the Italian Association of Hospital Surgeons, the Multi-specialist Italian Society of Young Surgeons, the Italian Society of Emergency Surgery and Trauma, the Italian Society of Anesthesia, Analgesia, Resuscitation and Intensive Care, the Italian Society of Orthopaedics and Traumatology, the Italian Society of Emergency Medicine, the Italian Society of Medical Radiology, Section of Vascular and Interventional Radiology and the World Society of Emergency Surgery. From November 2012 to January 2013 the SC undertook the critical revision and prepared the presentation to the audience and the Panel on the day of the Conference. Then 3 recommendations were presented according to the 3 submitted questions. The Panel voted the recommendations after discussion and amendments with the audience. Later on a email debate took place until December 2013 to reach a unanimous consent. We present results on the 3 following questions: which hemodynamically unstable patient needs an extraperitoneal pelvic packing? Which hemodynamically unstable patient needs an external fixation? Which hemodynamically unstable patient needs emergent angiography? No longer angiography is considered the first therapeutic maneuver in such a patient. Preperitoneal pelvic packing and external fixation, preceded by pelvic binder have a pivotal role in the management of these patients.

Hemodynamically Unstable Pelvic Trauma is a frequent death cause among people who sustain blunt trauma. We present the results of the First Italian Consensus Conference.

## Introduction

Hemodynamically unstable pelvic trauma is a major problem in trauma surgery and even in the most experienced Trauma Centers. A long living debate in the literature, with plenty of classifications and protocols, has not still established the best treatment strategy for these patients [1-6].

In recent years the EAST (Eastern American Society for Trauma) published the Management Guidelines on Hemorrhage from Pelvic Trauma which were developed by a named group of leading surgeons and physicians [6]. As in Italy this topic has never been faced in a public scientific debate, a National Consensus Conference (CC) was held in Bergamo on April 13th, 2013.

## Methods

An Organizing Committee (OC) from the Papa Giovanni XXIII Hospital of Bergamo [Italy] was established to organize a National Consensus Conference on Unstable Pelvic Trauma. Regulations in order to conduct the CC were adopted from “The Methodological Manual – How to Organize a Consensus Conference”, edited by the Higher Health Institute [7]. Levels of evidence (LoE) and grade of recommendations (GoR) come from Center for Evaluation of the Efficacy of Health Treatment (CeVEAS), Modena, Italy: six levels of evidence and five grade of recommendations have been defined (Table 1) [8]. A systematic review of the literature from 1990 to November 2012, commissioned by the OC, was undertaken by two reference librarians in December 2012. The electronic search was undertaken in following databases: MedLine, Embase, Cochrane, Tripdatabase, National Guidelines Clearinghouse, NHS Evidence, Trauma.org, Uptodate. In the meantime 9 Scientific Societies, both Italian and International, identified by the OC as among those interested in this topic, were asked to appoint 2 members each to participate in the CC organization. The following societies appointed the two requested members in December 2012: the Italian Society of Surgery (Società Italiana di Chirurgia, SIC), the Italian Association of Hospital Surgeons (Associazione dei Chirurghi Ospedalieri Italiani, ACOI), the Multi-specialist Italian Society of Young Surgeons (Società Polispecialistica Italiana dei Giovani Chirurghi, SPIGC), the Italian Society of Emergency Surgery and Trauma (Società Italiana di Chirurgia d’Urgenza e del Trauma, SICUT), the Italian Society of Anesthesia, Analgesia, Resuscitation and Intensive Care (Società Italiana di Anestesia, Analgesia, Rianimazione e Terapia Intensiva, SIAARTI), the Italian Society of Orthopaedics and Traumatology (Società Italiana di Ortopedia e Traumatologia, SIOT), the Italian Society of Emergency Medicine (Società Italiana di Medicina d’Emergenza-Urgenza, SIMEU), the Italian Society of Medical Radiology, Section of Vascular and Interventional Radiology (Società Italiana di Radiologia Medica, SIRM, Sezione di Radiologia Interventistica e Vascolare) and the World Society of Emergency Surgery (WSES).

**Table 1:** Levels of evidence and grade of recommendations

**Levels of evidence**	
**I**	RCTs and/or systematic review or metanalysis of RCTs
**II**	A single well designed RCT
**III**	Cohort studies with concurrent or historical controls or their metanalysis
**IV**	Case control studies or their metanalysis
**V**	Case series without controls
**VI**	Expert opinion, guidelines, documents coming from consensus conference
**Grade of recommendations**	
**A**	Highly recommended. From good quality level, even if not level I-II
**B**	Not always recommended but must be taken in consideration
**C**	Substantial uncertainty in favour or against
**D**	Not recommended
**E**	Highly not recommended

Among these societies’ delegates, the OC named the Scientific Committee (SC, 9 members) and the Jury Panel (JP, 9 members) in which each society was represented. The SC had the responsibility of creating 3 presentations according to the retrieved literature to answer the 3 questions selected by the OC.

The three questions were:

1. Which hemodynamically unstable patient needs a preperitoneal pelvic packing (PPP)?

2. Which hemodynamically unstable patient needs an external fixation (EF)?

3. Which hemodynamically unstable patient needs emergent angiography (AG)?

The OC reviewed the retrieved papers and selected the most appropriated as related to the three topics. Studies not directly addressing the management of hemodynamically unstable pelvic trauma were excluded (elective procedures, stable patients, reviews studies). Manual cross-reference search of the relevant studies was performed by the OC and the related relevant papers were also retrieved. The selected papers were subsequently sent to the members of the SC in late December 2012, helping in the review of the literature. The SC and the OC shared the presentation in late February and completed the work in early March 2013. At the conference was also invited a representative of a voluntary association the Italian Association of Blood Volunteers (Associazione Volontari Italiani del Sangue, AVIS), as a representative of the civil society. During the day of the conference (April 13 th 2013) the SC presented in the morning the whole review of the literature and in the afternoon the statements for each of the three questions. The JP, who was previously aware of the content of presentations and statements, discussed with the audience the results and formally approved the statements. Furthermore an algorithm for the whole management of hemodynamically unstable pelvic trauma was proposed during the conference. In the subsequent months the discussion took place by email and the overall content of the conference was definitely approved by all the members of the three committees. The Scientific Societies gave the last approval and permission for submission and publication.

## Results and discussion

**Figure 1:**
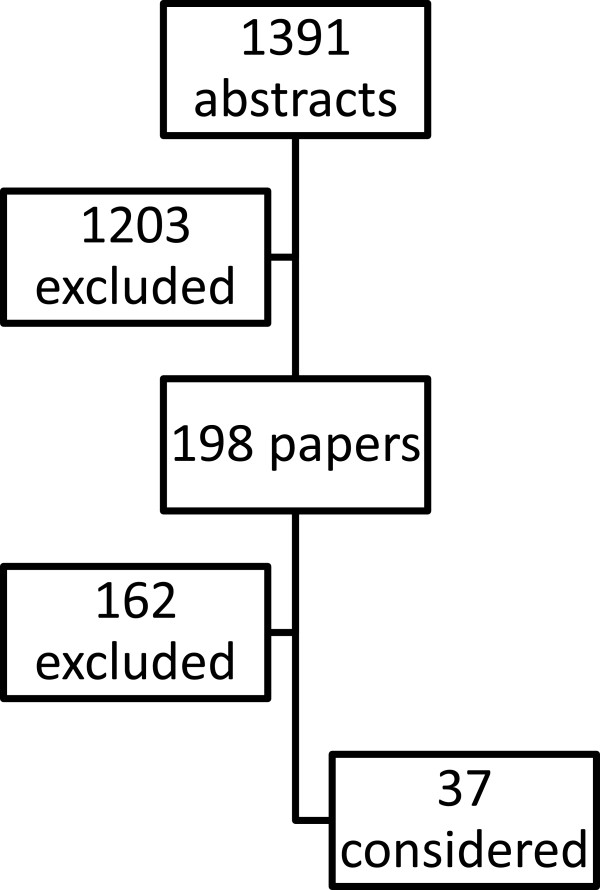
Bibliographical search.

The electronic search (Figure 1) gave 1391 abstracts. Of these 1203 were excluded (not directly related topic, stable patients, mixed population, elective procedures). Among the 198 remaining papers, 162 were excluded (elective procedures, overlapping data, stable patients, expert opinion, review). Finally 36 papers were considered (Table 2). No randomized controlled trials were found, but only case series and case-control studies. The SC presented this revision of the literature trying to answer the three previously decided topics at the conference day. This public conference was attended by 160 scientists and experts. Each revision was focused to answer one of the three questions and was followed by a public debate. During the lunch meeting the SC and the JP discussed the statements reaching an informal consensus and in the afternoon the statements were presented to the audience. The conference was closed after a public debate which strengthened the statements and produced a draft for an algorithm for the whole management of hemodynamically unstable pelvic trauma. Later on the SC and the JP, with the OC, discussed the algorithm via email and finally approved it. For the purposes of the CC we define hemodynamically unstable a patient which needs ongoing appropriate resuscitation without reaching a target systolic blood pressure of 90 mmHg and pelvic trauma is, together or not with other traumatic lesions, responsible for this hemodynamic status. Patient in extremis is a “bleeding to death” one, with profound refractory shock despite a timely and correct resuscitation. Pelvic mechanical stability is defined according to AO/OTA classification [9].

**Table 2:** Revised papers 1990-2013

	**Reference**	**Year**	**Design**	**Patients**	**Comments**
1	Burgess [1]	1990	Prospective	25 unstable	Acute external fixation and angio
2.	Flint [10]	1990	Prospective observational	60	Use of PASG, 37/60 had ORIF within 24 hrs, only 4 ext fix
3.	Latenser [11]	1991	Prospective with historical controls	18/19	Early defined as internal or external fixation within 8 hrs from arrival
4.	Broos [12]	1992	Retrospective	44 type B and C fractures	Immediate fixation
5.	Gruen [13]	1994	Retrospective	36 unstable	Angio and anterior urgent ORIF [within 2-3 days]
6.	Van Veen [14]	1995	Retrospective	9 unstable	Peritoneal packing, bilateral ligation of internal iliac artery, EF and/or ORIF within 6 hours
7.	Heini [15]	1996	Retrospective	18 unstable	C clamp placement
8.	Bassam [16]	1998	Prospective observational	15 unstable	External fixation first if anterior fracture, angio first if posterior fracture
9.	Velmahos [17]	2000	Retrospective	30 unstable	Bilateral embolization of iliac internal artery
10.	Wong [18]	2000	Retrospective	17 unstable	External fixation and angio, either before or after
11.	Biffl [19]	2001	Observational with historical controls	50/38 systolic blood pressure < 90	Use of angio and early external fixation or C clamp
12.	Ertel [20]	2001	Retrospective	20	Use of C clamp and pelvic packing
13.	Cook [21]	2002	Retrospective	74 unstable [23 underwent angio]	Exernal fixation and angio
14.	Kushimoto [22]	2003	Retrospective	29 mixed population	Angio before and after Damage Control Laparotomy. No pelvic packing or external fixation. High mortality.
15.	Miller [23]	2003	Retrospective	35 unstable	Angio and then external fixation. If laparotomy first angio done after external fixation
16.	Hagiwara [24]	2003	Prospective	61 stable and unstable	Angio and then external fixation in the angio suite
17.	Ruchholtz [25]	2004	Prospective	21 unstable	Early external fixation in mechanically unstable fractures
18.	Fangio [26]	2005	Retrospective	32 unstable	Angio first usually. No packing. Laparotomy before or after angio. Some external fixation
19.	Sadri [27]	2005	Retrospective	14 unstable	C clamp and then angio
20.	Krieg [28]	2005	Prospective	16 unstable	Outcomes following pelvic belt
21.	Croce [29]	2007	Retrospective	186 [stable and unstable]	Use of External fixation or T-POD® and angio
22.	Lai [30]	2008	Retrospective	7 unstable	External fixation and angio
23.	Richard [31]	2009	Prospective	24 APC-2 pelvic injuries [11 unstable]	Anteriorly placed C-clamp [in the ER, angio suite or OR]
24.	Morozumi [32]	2010	Retrospective	12 unstable	Mobile angio first. No packing or fixation
25.	Jeske [33]	2010	Retrospective	45 unstable	External fixation and angio
26.	Enninghorst [34]	2010	Retrospective	18 unstable	Acute ORIF [< 24 hrs]
27.	Tan [35]	2010	Prospective	15 unstable	Application of T-POD®
28.	Cherry [36]	2011	Retrospective	12 unstable	OR angio.
29.	Karadimas [37]	2011	Retrospective	34 mixed population	External fixation and secondary angio.
30.	Hornez [38]	2011	Retrospective	17 unstable	Pelvic packing, angio and fixation.
31.	Fang [39]	2011	Retrospective	76 unstable	Mixed population [60% unstable fractures]. Angio and/or laparotomy. No packing.
32.	Tai [40]	2011	Retrospective	24 unstable	Shift to pelvic packing and external fixation before angio
33.	Burlew [41]	2011	Prospective	75	Preperitoneal pelvic packing and external fixation in emergency. Secondary angiography
34.	Fu [42]	2012	Retrospective	28 unstable	Angio [available 24 hrs] directly if negative FAST. Intraperitoneal packing. No fixation.
35.	Hu [43]	2012	Retrospective	15 unstable	External fixation
36.	Metsemakers [44]	2013	Retrospective	98 unstable	External fixation first, no pelvic packing for closed fractures. Then angio [13 embolized out of 15 angio done]
37.	Abrassart [45]	2013	Retrospective	70 unstable	4 groups with either external fixation only, together with angio, laparotomy or angio before external fixation

Statements were approved as follow:

### Preperitoneal pelvic packing (PPP)

#### Background

In the last 10 years PPP has gained popularity as a tool to control venous bleeding in pelvic trauma. Since the first report from Pohlemann in 1994 [46] and Ertel in 2001 [20] many papers demonstrated this is a feasible, quick and easy procedure. PPP has been already adopted in some centers as a key maneuver for unstable patients [41]. It can be accomplished both in the emergency department (ED) and the operating room (OR). Our CC agreed that PPP can be quickly done both in the shock room in the ED or in the OR, according to local organization. In a mechanically unstable pelvic fracture PPP has to be done together with fixation of the pelvis with EF, when feasible and possibile, as indicated by Pohlemann [46], Ertel [20] and Cothren [47] as well as others authors [3,4,15,25,41,45]. In conclusion PPP is a pivotal procedure, as well as external stabilization, in the emergency setting, both in the OR and the ED. When patient is in extremis PPP, together with external stabilization can be life saving.

### Statements

1. PPP is effective in controlling hemorrhage when used as part of a multidisciplinary clinical pathway including AG and EF. **[GoR B, LoE IV]**

2. PPP is effective in controlling hemorrhage when used as a salvage technique. **[GoR B, LoE IV]**

### External fixation

#### Background

The volume of the pelvis increases after a mechanically unstable pelvic fracture. EF has always been the mainstay of emergency treatment in order to reduce the volume of the pelvis and control hemorrhage [46,48-50]. Two main techniques are available to externally fix the unstable pelvic ring: external fixator and C-Clamp. While the external fixator is indicated in type B fractures, the pelvic C-clamp is used in unstable C type injuries, according to AO/OTA classification [9].

Temporary binders are used to control the hemorrhage from the pelvic fractures. These devices are very simple and quick to apply, and they can reduce the pelvic volume. However pelvic binders (PB) are not external fixator because they do not provide mechanical stabilization of the pelvis and they must be removed within 24 hours to avoid pressure sores on the patient. The data confirming efficacy of pelvic binders in controlling hemorrhage from pelvic fracture remain unclear because of conflicting studies in the literature [28,29,51,52].

The Consensus Conference considered EF a pivotal procedure in presence of a mechanically unstable pelvic fracture and agreed that EF can be performed both in the shock room in the ED or in the OR, according to the local facilities. PB is a valid tool, mainly if applied in the prehospital setting, as a bridge to fixation. It can provide an external stabilization that could be life saving in patients in extremis. When EF is not possible (ie orthopedic surgeon is on call during night hours) PB is a valid alternative, provided EF is accomplished as soon as possible or the patient transferred to another facility.

### Statements

1. PB should be applied as soon as pelvic mechanic instability is assessed, better in the prehospital setting **[GoR A, LoE III]**

2. Anterior or posterior EF must be accomplished in unstable fractures as soon as possible in substitution of PB **[GoR B, LoE III]**

3. EF can be accomplished in the ED or in the OR and appear to be a quick tool to reduce venous and bony bleeding **[GoR A, LoE IV]**

4. EF, whenever possible, can be the first maneuver to be done in patients with hemodynamic instability and a mechanically unstable pelvic fracture **[GoR A, LoE IV]**

### Angiography

#### Background

AG emerged in the ‘80s as a valid tool to control arterial bleeding [53-55] and for many years has been regarded in the vast majority of trauma centers as the first-line treatment in unstable patients. On the other hand it has long activation time, as teams are often on call and they are not present in the hospital on a 24 hours basis. In the last years improvement of technology allowed for portable instruments [32,36] that can lower the threshold for indication towards this method.

### Statements

1. After non-pelvic sources of blood loss have been ruled out, patients with pelvic fractures and hemodynamic instability or signs of ongoing bleeding should be considered for pelvic AG/embolization. **[GoR A, LoE III]**

2. Patients with CT-scan demonstrating arterial intravenous contrast extravasation in the pelvis, may require pelvic AG and embolization regardless of hemodynamic status. **[GoR A, LoE III]**

3. After non pelvic sources of blood loss have been ruled out, patients with pelvic fractures who have undergone pelvic AG with or without embolization, with persisting signs of ongoing bleeding, should be considered for repeat pelvic AG/embolization **[GoR B, LoE IV]**

### The decisional algorithm

**Figure 2:**
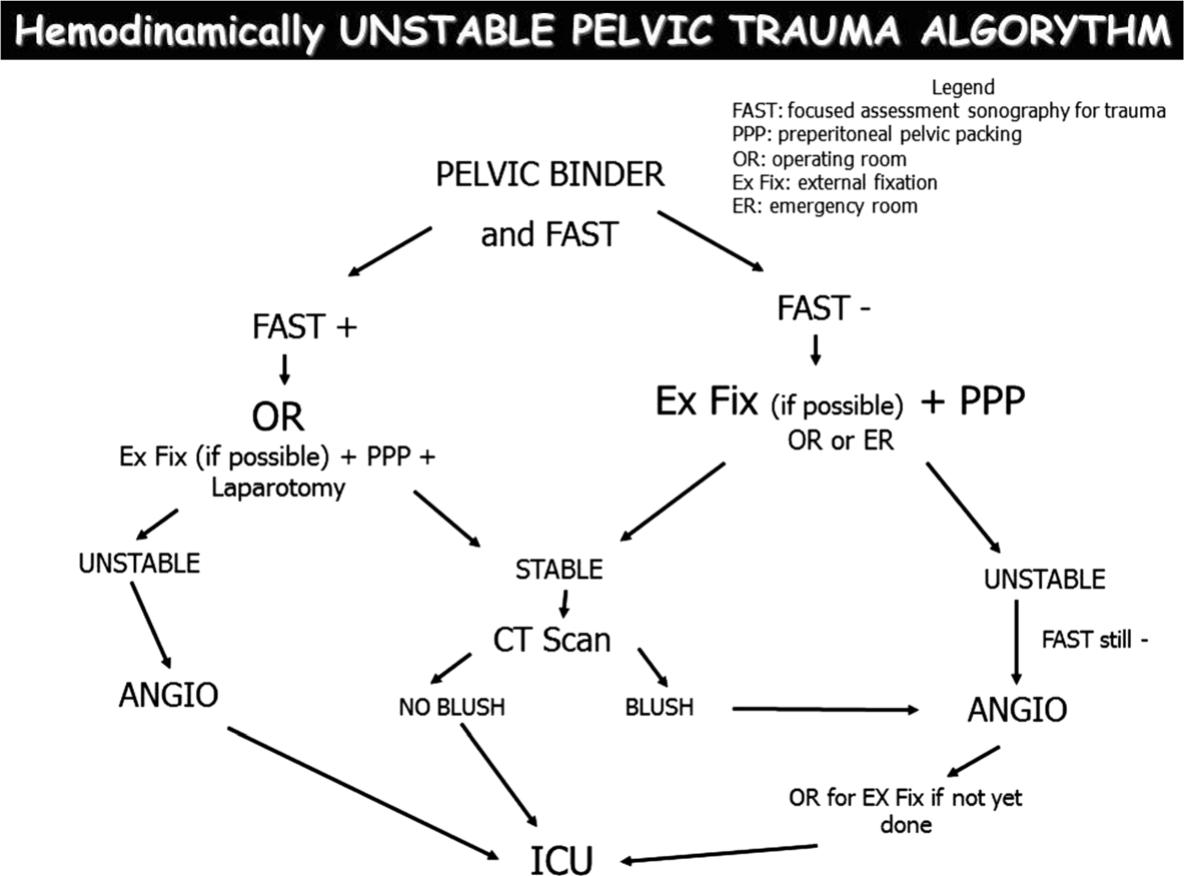
Treatment algorithm.

During the Conference, after debating the statements, a draft for an algorithm was proposed to the SC, the JP and the audience (Figure 2). A formal consensus was reached on the use of PPP, as a first maneuver only, in mechanically stable fractures of the pelvis. In mechanically unstable fractures EF should be applied as a substitution of the PB as soon as possible even in the ED or in the OR according to local protocols. PPP without any kind of mechanical stabilization is not adequate, because it needs a stable frame for packing to be effective.

In the last few months the algorithm was written in detail and conducted to a double pathway according to the local expertise/availability of trauma surgeons/orthopedics. In the unstable patient EF can be done in the ED or the OR. The unanimous consent in the Conference regards the fact that AG is no more considered the first maneuver in the unstable patient, but is considered only for patients who remains unstable after EF and PPP.

## Conclusions

Hemodynamically unstable pelvic trauma is a challenging task in most Trauma Centers. No unanimous consent is present in the literature regarding the best treatment for these patients. The First Italian Consensus Conference on this topic extensively reviewed the current available knowledge and proposed a readily available algorithm for different level and experience hospitals.

## Competing interests

The authors declare that they have no competing interests.

## Authors’ contributions

SM wrote the paper with the contribution of FC and LA. RM and DP helped in retrieving the papers in the literature and reviewed all of them. All the authors revised the paper and gave approval for submission and publication.
